# Dual Pathway Inhibition in Patients With mRCC: A Case Series From Kuwait Highlighting the Role of Avelumab and Axitinib

**DOI:** 10.1155/crom/8007226

**Published:** 2026-04-10

**Authors:** Mohamed Ashour, Mostafa El-Shahat

**Affiliations:** ^1^ Kuwait Cancer Control Center, Sabah Health Area, Kuwait City, Kuwait, kuwaitcancercenter.net; ^2^ Al-Azhar University Faculty of Medicine for Boys, Cairo, Egypt

**Keywords:** avelumab, axitinib, dual pathway inhibition, Kuwait, metastatic, renal cell carcinoma

## Abstract

**Background:**

Renal cell carcinoma (RCC) accounts for approximately 3% of adult cancers and is characterized by aggressive behavior and early metastatic potential. The therapeutic paradigm of metastatic RCC (mRCC) has evolved from tyrosine kinase inhibitors (TKIs) alone to immune checkpoint inhibitors (ICIs) and ICI–TKI combinations. Avelumab, an anti–PD‐L1 antibody, in combination with axitinib, a VEGFR‐targeted TKI, has demonstrated efficacy in pivotal trials such as JAVELIN Renal 101.

**Objective:**

The objective of this study is to report the clinical outcomes of four patients with advanced mRCC and extensive metastases treated with avelumab with axitinib at the Kuwait Cancer Control Center (KCCC).

**Methods:**

This case series describes diagnostic imaging, treatment regimens, and disease progression in four patients. Imaging modalities included PET‐CT and CT scans, performed at multiple intervals to assess therapeutic response, disease control, and treatment tolerability.

**Results:**

All patients demonstrated favorable radiological and clinical responses despite high metastatic burden and the presence of comorbidities, including disease regression or stable disease across multiple metastatic sites. Treatment was generally well tolerated, with no Grade III or IV toxicities recorded.

**Conclusion:**

This case series involving patients treated at KCCC demonstrates the real‐world efficacy and tolerability of avelumab plus axitinib as a first‐line option for metastatic renal cell carcinoma, even in patients with high metastatic burden, comorbidities, or prior treatment failure. Outcomes observed are consistent with JAVELIN Renal 101 trial findings, reinforcing the therapeutic value of dual immune and angiogenic pathway inhibition in advanced RCC care. Further investigation into biomarkers and patient selection may help optimize clinical outcomes across diverse populations.

## 1. Introduction

Renal cell carcinoma (RCC) is a primary malignancy of the kidney, representing the most common form of kidney cancer. [1] Known for its aggressive nature and potential for early metastasis, RCC is often challenging to detect in early stages due to its asymptomatic development. When RCC spreads, it frequently affects the lungs, liver, adrenal glands, bones, and lymph nodes. Due to its high metastatic potential, RCC is associated with poor survival outcomes, especially in advanced stages. RCC accounts for approximately 3% of all adult cancers. It is a tumor of the older age group and is mostly seen between the ages of 50 and 70 years, with an approximately 2:1 male‐to‐female ratio [1].

Each year, the global incidence of RCC continues to rise, partly due to improved imaging techniques but also due to increased risk factors such as smoking, hypertension, and obesity. [2] When RCC is diagnosed at an early stage, surgical intervention can be pursued with the aim of achieving a cure. However, for cases identified at an advanced or metastatic stage, active surveillance may be considered if the metastases are growing slowly. Nonetheless, most instances will lead to disease progression, necessitating systemic therapy. Traditionally, the first‐line treatment for metastatic renal cell carcinoma (mRCC) involved the use of tyrosine kinase inhibitors (TKIs) that target the vascular endothelial growth factor receptors (VEGFR). Recently, the treatment landscape for first‐line therapy has evolved dramatically due to the ground‐breaking introduction of immune checkpoint inhibitors (ICIs) and combinations of VEGFR TKIs with ICIs, ushering in a new era for RCC management. [3] Dual immune checkpoint blockade with nivolumab plus ipilimumab has demonstrated durable responses and overall survival benefit, particularly in patients with intermediate‐ and poor‐risk disease. [4] However, antiangiogenic therapies that focus on VEGF and its receptors (VEGFRs) have provided significant benefits for patients with advanced RCC. The selection of avelumab in combination with axitinib for the patients described in this case series was based on multiple clinical considerations. All patients presented with extensive metastatic disease, requiring a treatment approach capable of inducing a rapid and meaningful tumor response. Axitinib, a selective VEGFR‐TKI, is approved for use as a second‐line monotherapy in cases of advanced RCC. Moreover, ICIs have exhibited antitumor effects in this context. Avelumab, a human IgG1 monoclonal antibody that binds to PD‐L1, prevents its interaction with PD‐1, showcasing the potential of this approach. The combination of an ICI with a TKI has emerged as a standard first‐line strategy for advanced RCC patients, offering a promising and effective treatment option. The effectiveness and safety of administering avelumab alongside axitinib as first‐line therapy for advanced RCC were initially demonstrated in the JAVELIN Renal 100‐phase Ib trial. Following the findings of the JAVELIN Renal 101 Phase III trial, the combination of avelumab and axitinib has received approval as the first‐line treatment for advanced RCC in several countries around the globe [5].

According to the latest health statistics, cancer is identified as the second leading cause of death in Kuwait, with an incidence rate of 21.7 per 100,000 population, surpassed only by cardiovascular diseases at 58.6 per 100,000 [6]. In 2015, Kuwait recorded 2700 new cancer cases, with 1319 cases among Kuwaitis and 1381 among non‐Kuwaitis. As of 2022, the 5‐year prevalence of kidney cancer in Kuwait was reported at 6.9 per 100,000. [7] Although RCC is not among the Top 10 most common cancers in the country, understanding its epidemiology within the Kuwaiti population and assessing treatment outcomes remain essential [6].

This case report presents four case studies of RCC patients with extensive metastasis who were treated with avelumab and axitinib. These patients were treated in the Kuwait Cancer Control Center (KCCC). The report details the patients′ clinical progression, diagnostic imaging findings, treatment approach, and response, discussing the challenges and potential benefits of this combination therapy in managing an extensive metastatic burden.

## 2. Case Report

### 2.1. Case 1: A Case of Stage IV mRCC With Metastasis to Lymph Nodes, Liver, and Lungs

A 68‐year‐old female with a history of clear cell RCC presented with Stage IV disease, metastatic to the lungs, liver, and lymph nodes. Her medical history included comorbidities of hypothyroidism, hypertension, and a skin allergy to nuts.

**Figure 1:**
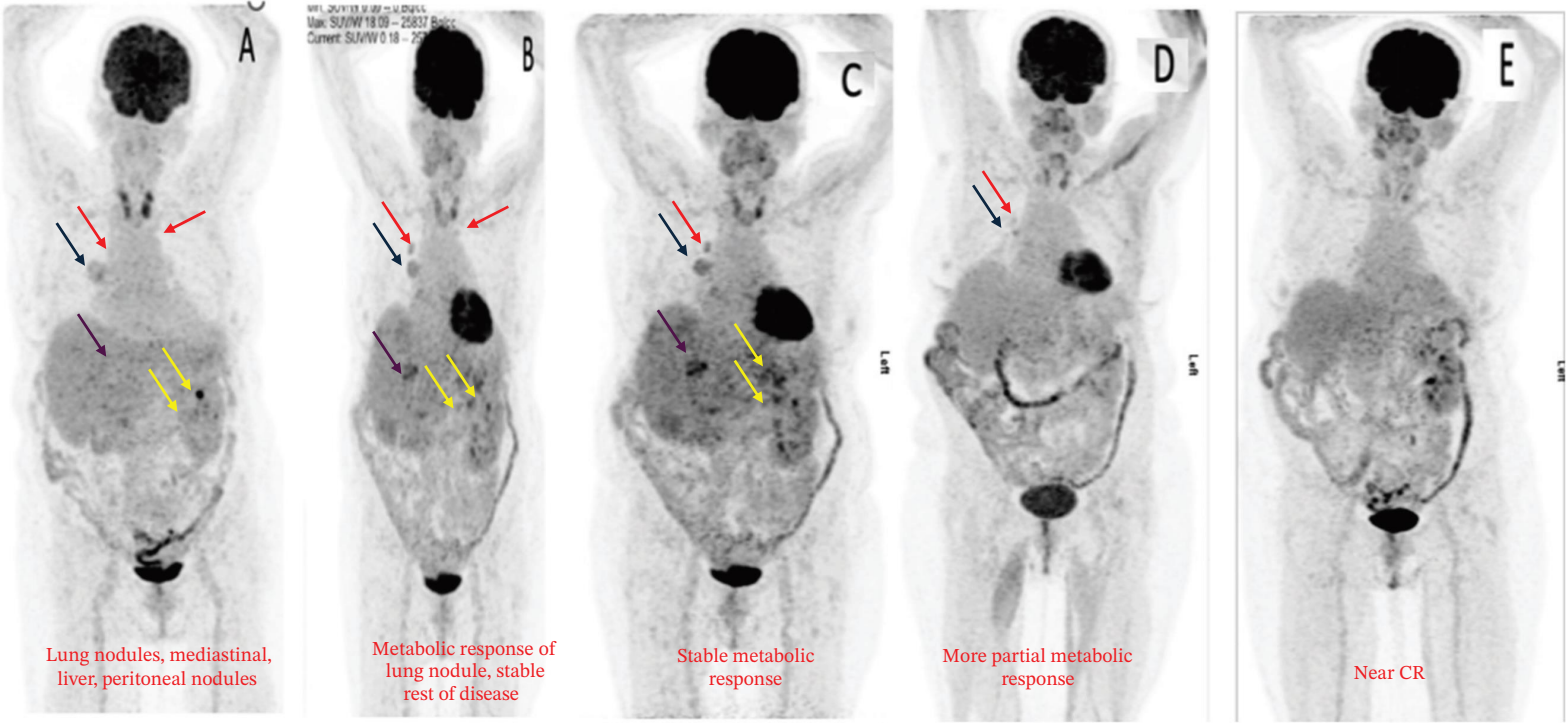
PET‐CT scans in (A) July 2023, (B) October 2023, (C) February 2024, (D) July 2024, and (E) April 10, 2025 (blue arrows: mediastinal nodes; orange arrows: pulmonary nodules; red arrows: liver nodules; and yellow arrows: peritoneal nodes).

Although the patient was in Syria in 2015, she was initially diagnosed with right‐sided RCC and underwent a right radical nephrectomy. Histopathology confirmed clear cell carcinoma, Grade 2, with an 11 cm tumor (pT3a N0 Mx). Following surgery, she was kept under routine surveillance. In November 2020, a positron emission tomography‐computed tomography (PET‐CT) scan identified a retroperitoneal mass and pulmonary nodules, suggesting metastatic disease. Fine needle aspiration cytology (FNAC) confirmed well‐differentiated clear cell carcinoma of renal origin. She had intermediate risk IMDC scoring and subsequently received multiple lines of therapy till May 2022, which included sunitinib, pazopanib, sorafenib, everolimus, and gemcitabine. A follow‐up computed tomography (CT) scan in June 2022 showed progressive liver, lung, mediastinal, and retroperitoneal lymph nodes. The patient was initiated on axitinib 5 mg BD. In January 2023, she presented to KCCC with ECOG 1 and controlled hypertension on a single antihypertensive medication. She had elevated renal function without radiological evidence of obstruction. Compared with the previous PET‐CT scan, there was size progression of the liver and mediastinal nodal disease, prompting a multidisciplinary decision. As the patient was not exposed to immunotherapy and avelumab could be accessed through the support program, she started combination therapy consisting of avelumab 800 mg IV every 2 weeks along with oral axitinib 5 mg twice a day. In April 2023, the patient tolerated avelumab and axitinib. Despite an initial rise, the serum creatinine levels improved with hydration, and the nephrologist changed her antihypertensive medications. The follow‐up PET‐CT scan showed stable size and metabolic activity of the liver, lung, mediastinal, and retroperitoneal nodal disease with no evidence of new lesions. On July 27, 2023, PET‐CT evaluation showed further mild regression in liver lesion size with stable metabolic activity in the lungs, mediastinal, and retroperitoneal lymph nodes. The patient remained clinically stable with no Grade 3 toxicities (Figure 1A). On October 29, 2023, the PET‐CT scan showed size regression in the lung nodules with stable size and metabolic activity of the liver, mediastinal, and retroperitoneal lymph nodes. There was no evidence of new hypermetabolic lesions. She had excellent tolerability to the treatment. On February 11, 2024, a follow‐up scan showed stable disease with no recorded toxicities (Figure 1C). On July 14, 2024, the PET‐CT scan showed partial metabolic response in the lung, mediastinal, and retroperitoneal disease with stable liver disease (Figure 1D). On December 30, 2024, the PET‐CT scan showed metabolic response in the liver, lungs, mediastinal, and retroperitoneal lymph nodes with no new lesions. The laboratory reports were satisfactory, allowing the patient to continue avelumab and axitinib with regular monitoring. The patient completed 2 years of avelumab treatment at the end of January 2025. She tolerated both avelumab and axitinib well with no recorded Grade III or IV toxicities (Figure 1E). On April 10, 2024, PET‐CT showed stable low metabolic activity in the liver, lungs, mediastinal, and retroperitoneal lymph nodes with no new lesions, allowing her to continue avelumab and axitinib with regular monitoring. She tolerated the treatment well with no recorded Grade III or IV toxicities.

### 2.2. Case 2: A Case of Stage IV RCC (Poor Risk) With Metastasis to the Lymph Nodes, Liver, Lungs, and Bone

A 56‐year‐old male with no history of comorbidities, allergies, or familial cancer, presented with right loin and back pain in March 2024. On April 13, 2024, an abdominal ultrasound identified a solid lesion (3.8 × 3.5 cm) at the right renal upper pole, alongside lymphadenopathy in the hepatorenal and paraaortic regions. The largest lymph node measured 2.1 cm. On April 24, 2024, a CT scan of the chest, abdomen, and pelvis showed a right renal mass associated with multiple enlarged paraaortic soft tissue masses. Metastatic spread was observed involving the liver, lung, mediastinal lymph nodes, and extensive skeletal (bone) structures (Figure 2). On May 1, 2024, a core needle biopsy confirmed clear cell RCC Grade 3. On May 15, 2024, a PET‐CT scan revealed extensive metastasis, showing a large hypermetabolic right renal mass with adjacent adrenal and retroperitoneal lymph nodes (SUV max 18.4). Hepatic involvement included two hypermetabolic lesions in the dome of the liver (SUV max 5.6) and Segment VII (SUV max 8.7), as well as left adrenal gland hypermetabolism (SUV max 9.5). Extensive bone metastases were further observed, especially in vertebrae T7, T11, L1, L2, L5, and S3, with high SUV values up to 12.8. A large hypermetabolic left supraclavicular lymph node mass was observed with a hypermetabolic lesion in the left lower lung lobe. Multiple hypermetabolic pulmonary nodules were scattered across both lungs, with the highest activity in the right basal segment. There was hypermetabolic bilateral mild pleural effusion (more on the left side) and hypermetabolic mediastinal lymph nodes, reaching a maximum SUV of 18.1.

Figure 2(a) CT scan on April 24, 2024, (b) PET‐CT scan on May 15,2024, (c) PET‐CT scan in September 2024, (d) PET Scan on December 26, 2024, and (e) PET scan on March 26, 2025.

**Figure:**
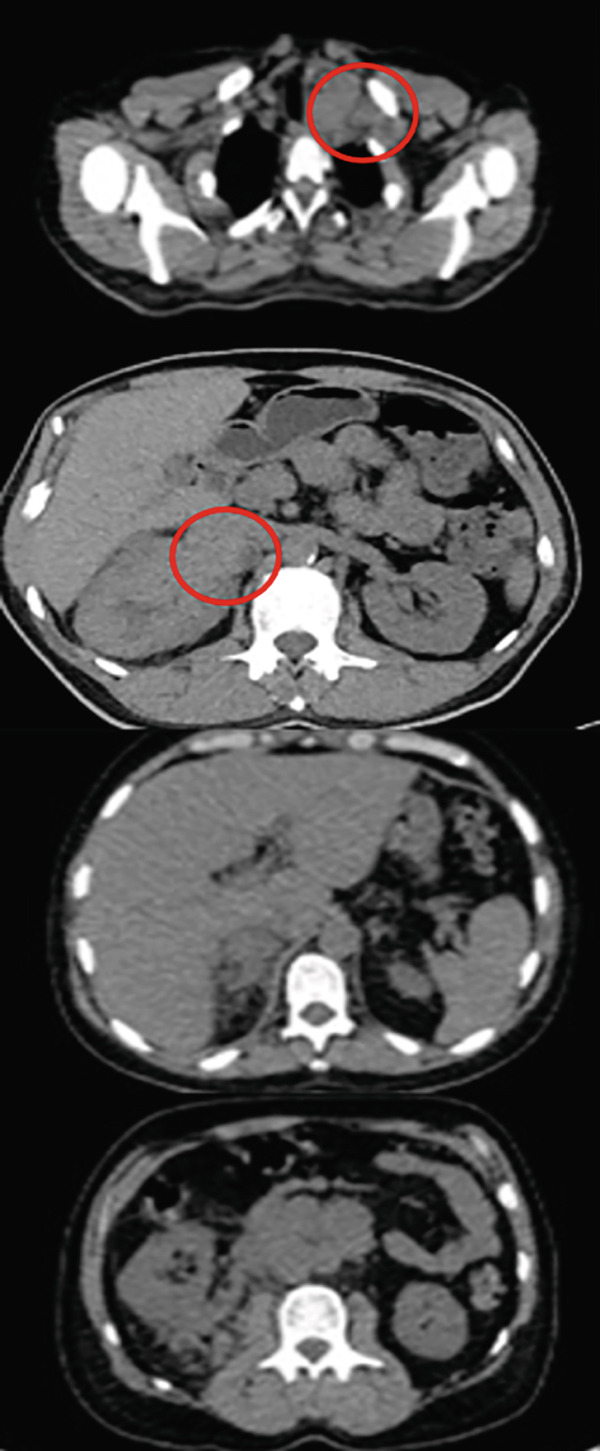
(a)

**Figure:**
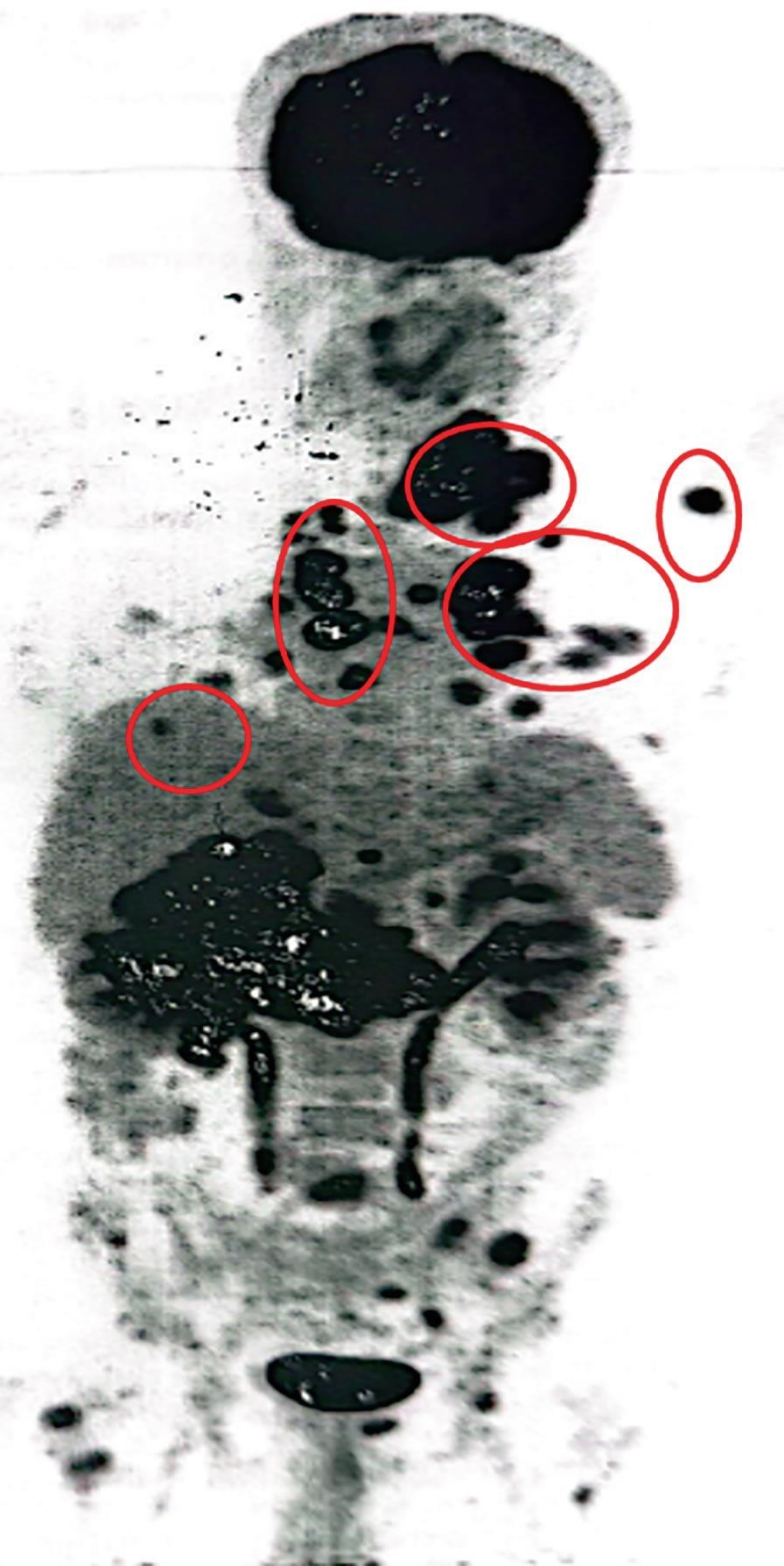
(b)

**Figure:**
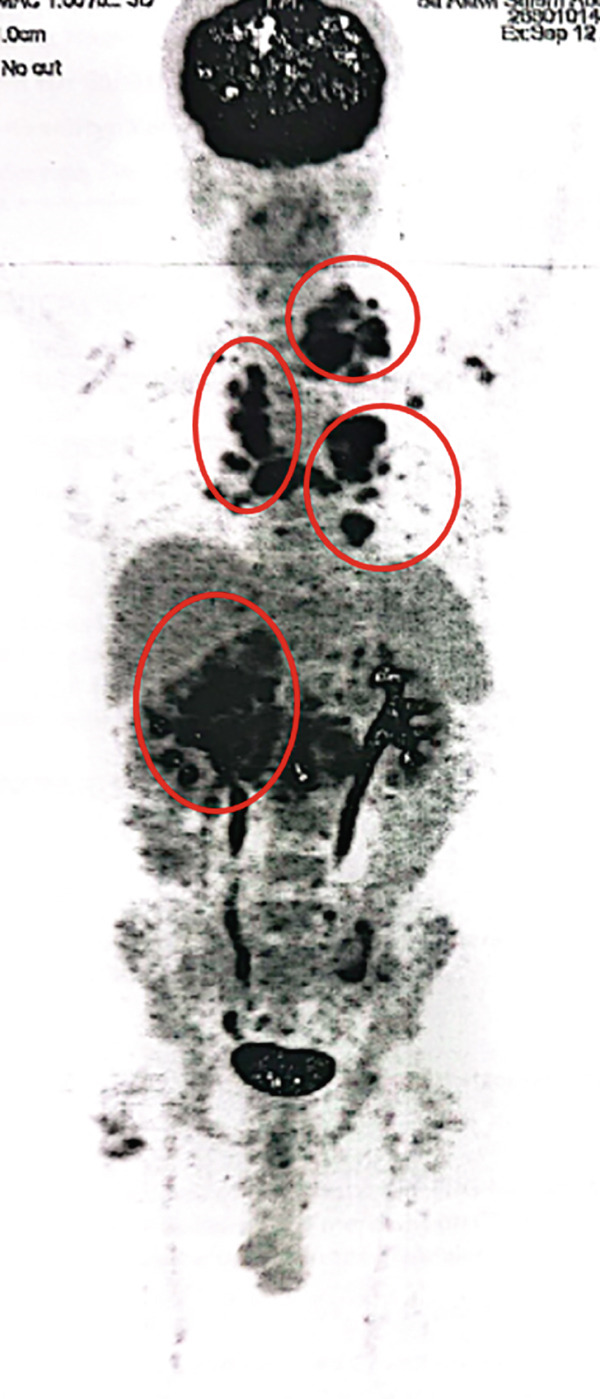
(c)

**Figure:**
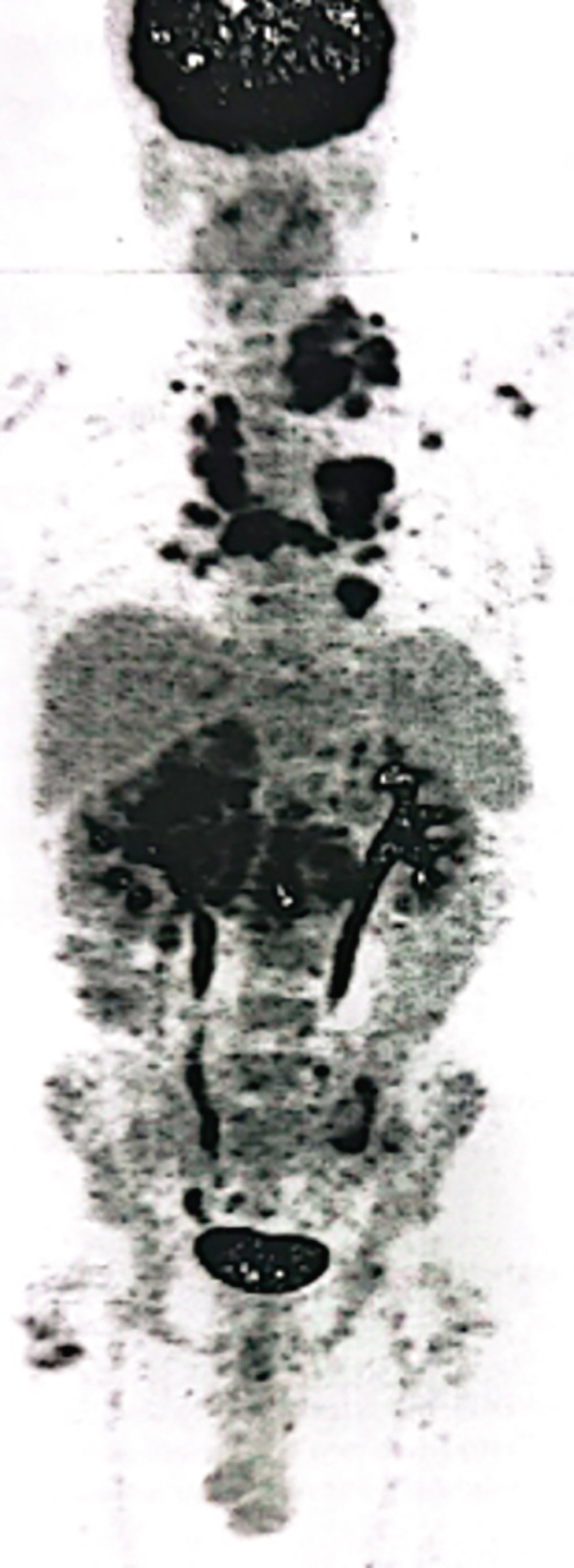
(d)

**Figure:**
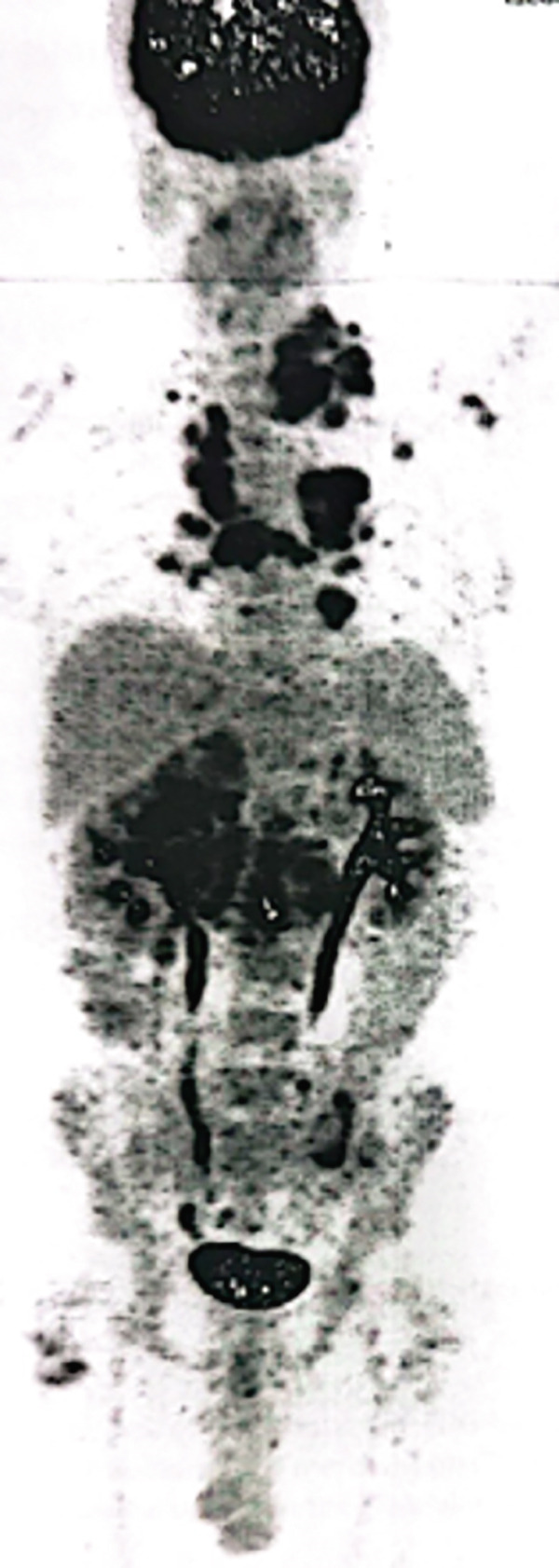
(e)

On June 13, 2024, the patient was ECOG 1 with satisfactory laboratory test outcomes (poor risk IMDC scoring) and mild back pain controlled on simple analgesics without adding narcotics. He was started on avelumab 800 IV every 2 weeks, axitinib 5 mg BD, and denosumab 120 mg SC every 28 days. In August 2024, the patient showed clinical improvement in his back pain with less use of analgesics. However, he developed Grade 1 hypertension, which was controlled by a single‐agent antihypertensive treatment, and had Grade 1 hepatitis. In September 2024, the follow‐up PET‐CT scan demonstrated size and metabolic regression of all metastatic sites, particularly in the lymph nodes and bones, suggesting a positive response to treatment without Grade 3 toxicities. On December 26, 2024, a PET scan showed stable disease with satisfactory laboratory reports. The patient had stable clinical conditions on avelumab plus axitinib, and the treatment was continued. On March 26, 2024, the PET scan continued to show stable disease with no new lesions. The patient also had satisfactory laboratory reports and was clinically stable on avelumab plus axitinib.

### 2.3. Case 3: A Case of Stage 4 RCC (Intermediate Risk) With Metastasis to Lymph Nodes and Lungs

In May 2023, a 50‐year‐old male with a medical history of long‐standing diabetes mellitus, CKD, ischemic heart disease (with stenting done 2 years ago), and hypertension presented with hematuria and weight loss. A CT scan of the kidneys, ureters, and bladder revealed a mass in the mid and lower poles of the right kidney (8 × 7.5 cm), involving perinephric fat and extending into the renal pelvis. There was irregular thickening of the bladder wall, whereas the chest CT was clear of metastasis. On May 31, 2023, the patient underwent a right open radical nephrectomy. Histopathology confirmed Grade 4 clear cell RCC, with tumor emboli in the renal vein and infiltration into renal sinus fat. Margins were clear, and lymph nodes (0/5) were negative for malignancy, staging the tumor as pT3N0. The patient had no access to adjuvant immunotherapy. On October 19, 2023, MRI of the abdomen showed an increase in the size of the left paraaortic lymph node to 1.5 × 1 cm, suggesting possible metastatic disease. A follow‐up scan on December 10, 2023, showed hypermetabolic activity in a right lower paratracheal lymph node (SUV max 7.1) and a nodule in the right upper lung lobe (SUV max 5) (Figure 3a). Multiple small pulmonary nodules were detected bilaterally without metabolic activity. There was significant metabolic activity in a paraaortic lymph node (SUV max 8) at the L3 level and a pericaval lymph node at the level of the right renal vessels (SUV max 3.7). On January 3, 2024, he underwent endobronchial ultrasound (EBUS) with needle aspiration from the paratracheal lymph node, which was positive for metastatic carcinoma, supporting the diagnosis of metastatic RCC. The patient had a status of ECOG 1 with elevated renal function and controlled blood pressure on an antihypertensive combination. His diabetes was also well controlled with oral medications. He was initiated on avelumab 800 mg IV every 2 weeks along with axitinib 5 mg twice daily.

Figure 3PET‐CT scans in (a) December 2023, (b) March 2024, (c) July 2024, (d) November 2024. (blue arrows: para‐tracheal LNS; orange arrows: pulmonary nodules; red arrow: paraaortic LNS).

**Figure:**
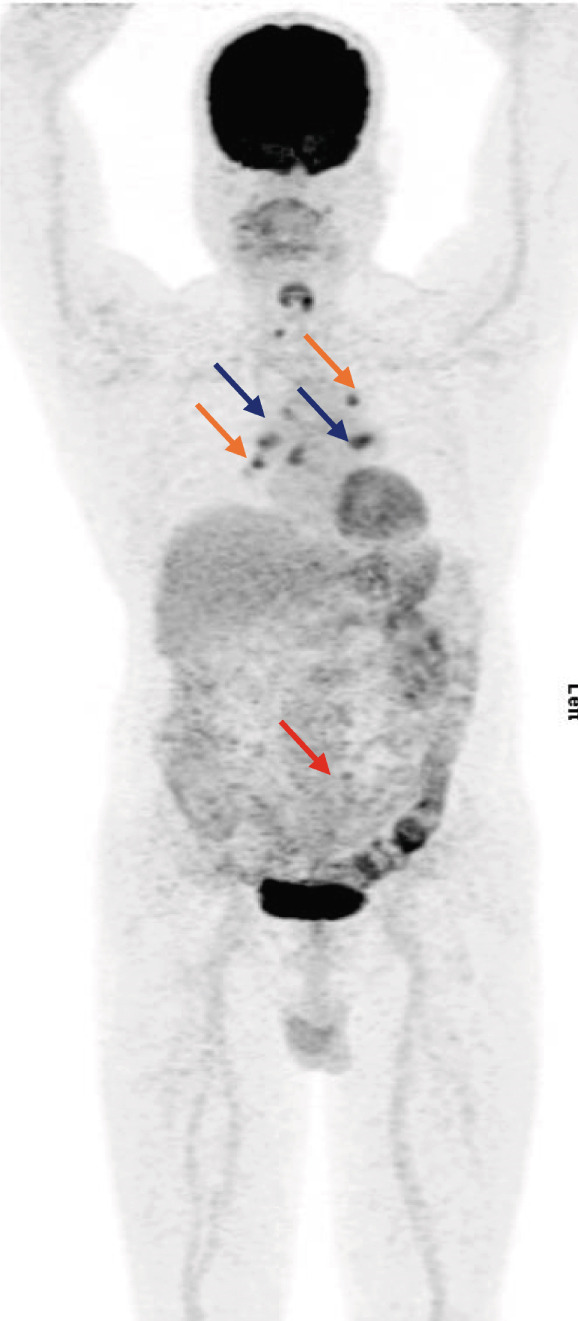
(a)

**Figure:**
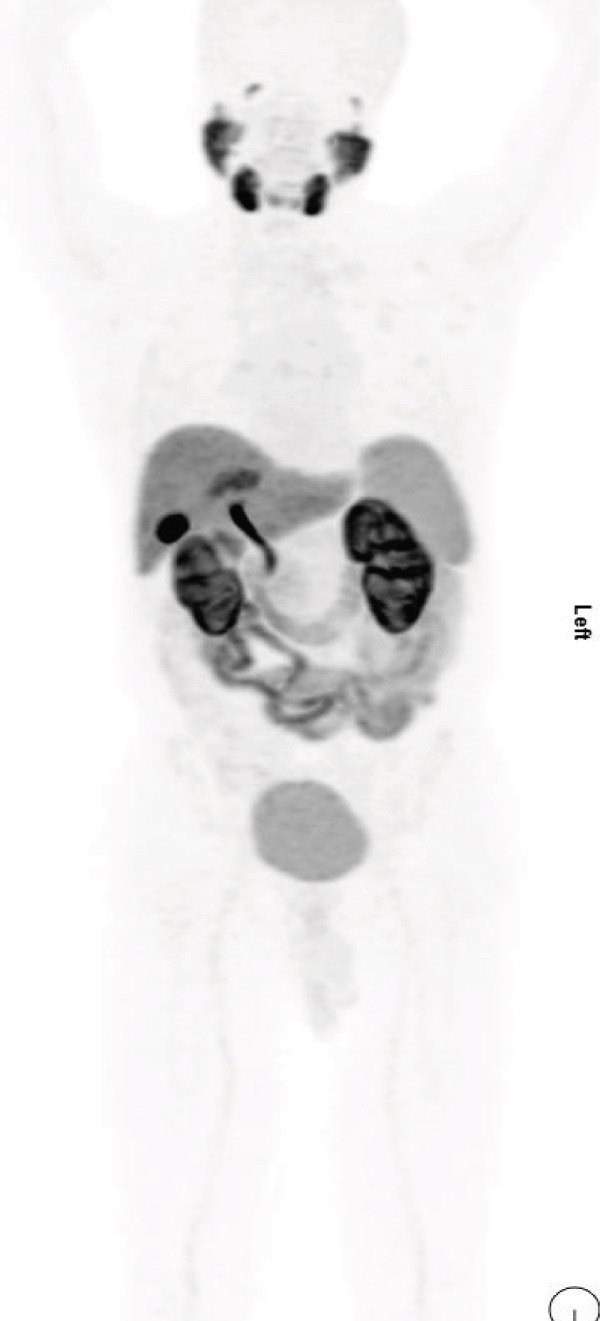
(b)

**Figure:**
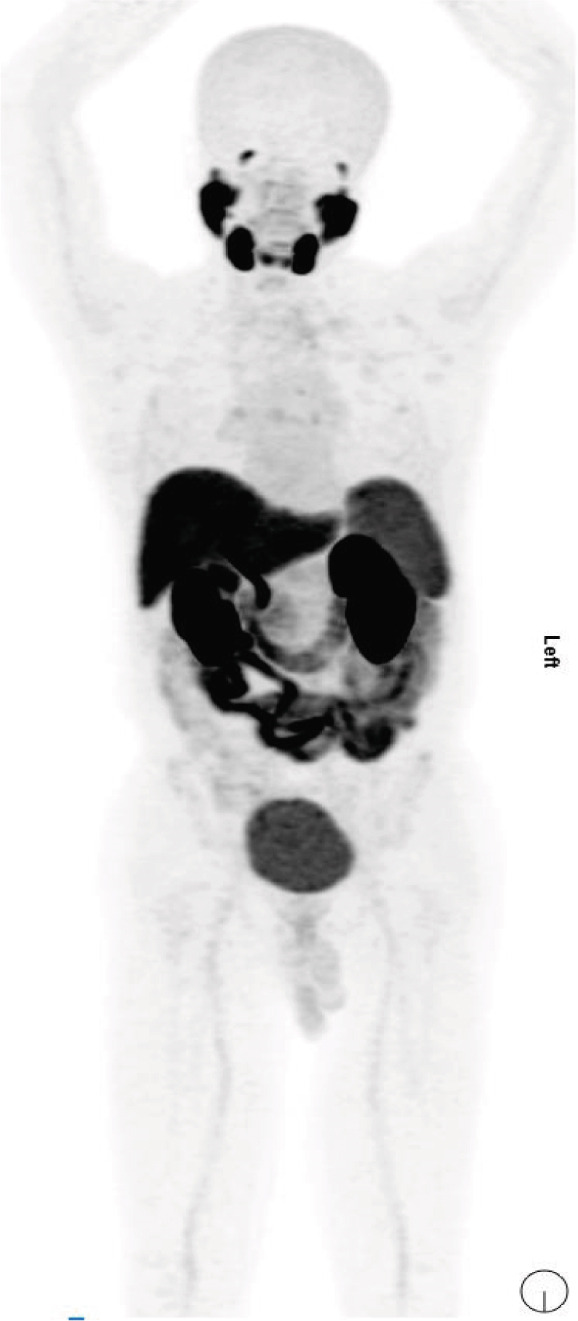
(c)

**Figure:**
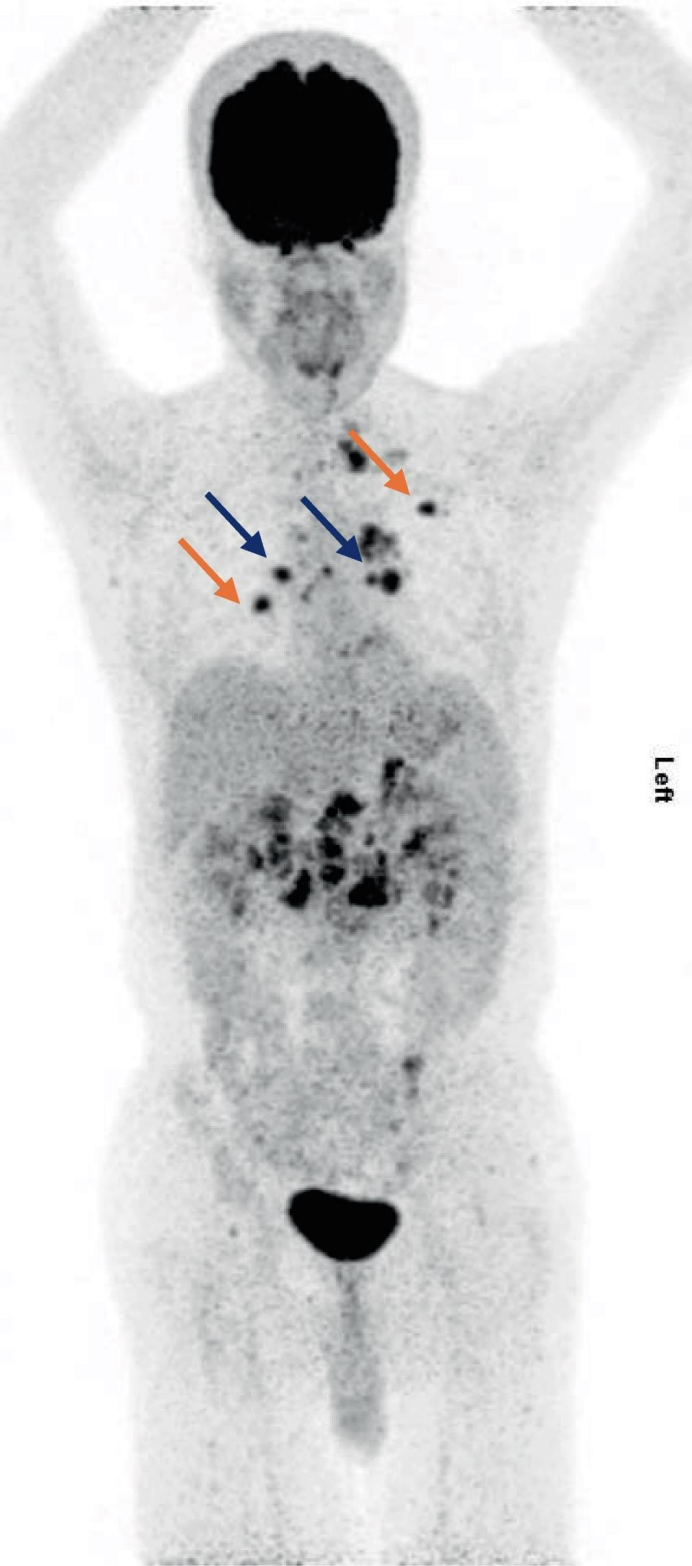
(d)

**Figure 4:**
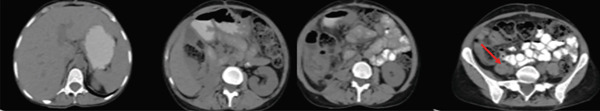
PET‐CT scans on November 18, 2023.

In March 2024, a follow‐up PET CT scan showed a resolved right upper lung nodule, right paratracheal lymph node, and marked metabolic response of the paraaortic lymph node. Stable bilateral hilar lymph nodes (SUV max 5.9) were observed with no recorded toxicities (Figure 3b). On July 4, 2024, the PET CT scan demonstrated that the patient continued to show good response with regards to the resolved right upper lung nodule, right paratracheal lymph node, and paraaortic lymph node. Stable bilateral hilar lymph nodes (SUV max 5.9) were observed, and the patient appeared to be tolerating the treatment well with no recorded toxicities (Figure 3c). On November 26, 2024, the PET‐CT scan showed the size and metabolic progression of bilateral hilar LNS with SUV (16.8 vs. 4.9 SUV). Newly enlarged hypermetabolic mediastinal nodal disease was observed. Chest CT showed newly developed bilateral metastatic lung nodules (Figure 3d). On January 6, 2025, the patient was prescribed second‐line cabozantinib. In March 2025, the PET‐CT scan showed size and metabolic regression of mediastinal and bilateral hilar nodal disease, with Grade 1 palmar–plantar erythrodysesthesia. The patient tolerated cabozantinib well and continued it.

**Figure 5:**
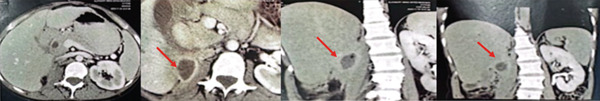
PET‐CT scans on March 17, 2024.

**Figure 6:**
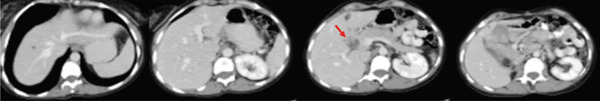
PET‐CT scans in April 2025.

### 2.4. Case 4: Stage IV Clear Cell RCC With Peritoneal Metastasis (Intermediate Risk)

A 47‐year‐old female without any comorbidities complained of abdominal pain and distention in mid‐September 2023. On September 27, 2023, the CT scan showed a large, exophytic mass in the right kidney upper lobe (14.4 × 12 × 9.4 cm), identifying the adjacent structure (liver, GB, parts of small bowel). The mass indented into superior mesenteric veins (SMV) and inferior vena cava (IVC) with no filling defect. There was engorgement of the mesenteric veins, thickening (omental cake) with multiple peritoneal nodules and ascites consistent with peritoneal metastases. On October 11, 2023, the patient underwent radical nephrectomy. Histopathology showed clear cell renal carcinoma (G4) with a tumor size of 16 × 15.5 × 3.5 cm. The tumor invaded the renal vein and sinus, reaching Gerota′s fascia, with focal sarcomatoid and rhabdoid features. The margins were involved with invasive carcinoma. Omental metastases were identified (omental fat involved) with positive ascitic fluid.

On November 18, 2023, the postoperative CT scan showed postsurgical inflammatory changes associated with tiny peritoneal nodules (Figure 4).

On January 7, 2024, she was ECOG 1 with satisfactory labs and was advised to start avelumab 800 IV every 2 weeks plus axitinib 5 mg BD. On March 17, 2024, the CT scan showed resolution of the previous surgical bed, small collection. The patient had mild free pelvic fluid and two necrotic peritoneal lesions (largest 2.5 cm) (Figure 5). She was ECOG 1 and tolerated the treatment well with no associated toxicities. On July 25, 2024, computed tomography–chest, abdomen, and pelvis (CT‐CAP) showed resolved ascites with stable two necrotic lesions and a peritoneal lesion at the gall bladder fossa. The patient continued on avelumab plus axitinib with no associated toxicities. On October 27, 2024, CT‐CAP showed stable disease as two stable necrotic peritoneal lesions at the gall bladder fossa. She was regular on her treatment with no associated toxicities and had satisfactory laboratory outcomes. On December 31, 2024, CT‐CAP showed a stable necrotic peritoneal lesion at the gall bladder fossa with no ascites or newly developed lesions. However, the patient complained of right upper abdominal pain. She had excellent tolerability and continued the same treatment. In April 2025, postdiscussion with the MDT on frequent upper abdominal pain attacks, she underwent exploration and excision of peritoneal disease. Histopathology showed metastatic carcinoma of clear cell renal origin (Figure 6), and the patient was advised to continue the same treatment.

## 3. Discussion

The case series highlights the role of the combination of avelumab and axitinib in patients with mRCC, aligning well with clinical trial data, particularly the findings from the JAVELIN Renal 101 study. This pivotal trial established avelumab plus axitinib as an effective first‐line treatment for mRCC, demonstrating significant improvements in progression‐free survival (PFS) compared with sunitinib, the previous standard of care [5] Each of the cases discussed in this series exhibited either disease stabilization or a partial response with the combination therapy, mirroring the benefits observed in clinical trials.

The first case illustrated the positive impact of combination therapy in a patient with an aggressive disease course, as evidenced by high activity in multiple lymph nodes and pulmonary nodules on PET scans. Treatment with avelumab plus axitinib led to disease control, closely reflecting outcomes seen in the JAVELIN Renal 101 trial. In that study, the combination therapy was particularly effective in patients with a high metastatic burden and aggressive disease, with a median PFS of 13.8 months in the overall population and 17.1 months in PD‐L1–positive patients. [8]. Similarly, the patient in this case experienced a reduction in nodal and pulmonary metastases, supporting the efficacy of dual pathway inhibition in managing high‐risk mRCC with a significant tumor burden. These findings are further supported by data from the J‐DART2 trial, the largest retrospective study reporting long‐term real‐world outcomes in Japanese patients with advanced RCC treated with avelumab and axitinib [8].

Additionally, the patient in the first case was 68 years old. His positive response aligns with extended follow‐up data from the JAVELIN Renal 101 trial, which confirmed the combination′s safety and efficacy across age groups, including elderly patients (≥ 65 years). Among patients aged 65–74 years, avelumab plus axitinib demonstrated improved PFS and higher tumor response rates compared with sunitinib. [9] Importantly, patients aged 75 years or older also benefited from the combination therapy, without a significant increase in adverse events compared with younger patients. Consistent with these findings, the elderly patient in this case responded favorably to treatment.

In the second case, the patient had previously received multiple lines of targeted therapies, including pazopanib, sorafenib, everolimus, and sunitinib, but experienced disease progression on each. Although the JAVELIN Renal 101 trial primarily assessed first‐line treatment, this case demonstrates the potential utility of avelumab and axitinib in later lines of therapy. Disease stabilization and partial responses observed in this case are particularly significant given the limited treatment options available for refractory RCC patients.

The third case involved a patient with high‐grade RCC and multiple comorbidities. Although no trials to date have specifically evaluated the efficacy of the combination therapy in such complex patients, this case showed that treatment with avelumab and axitinib resulted in disease stabilization. Notably, the patient responded despite experiencing treatment delays, suggesting a durable response. This observation is consistent with clinical trial findings, where disease control was maintained over time even with temporary interruptions due to adverse effects or logistical issues.

Although avelumab and axitinib were effective in providing disease control in all three cases, challenges such as medication access delays and treatment‐related adverse effects highlight the need for optimized management protocols to enhance adherence and continuity of care. Furthermore, although PD‐L1 positivity has shown predictive value in some contexts, its role in guiding response to combination therapy remains incompletely understood. Future studies should focus on exploring biomarkers such as PD‐L1 and tumor mutation burden to identify patients most likely to benefit from combination therapies like avelumab and axitinib.

## 4. Conclusion

This case series involving patients treated at KCCC demonstrates that avelumab and axitinib combination therapy is an effective treatment option for mRCC, particularly in cases with high metastatic burden, treatment resistance, and comorbidities. The results align with the JAVELIN Renal 101 trial findings, reinforcing the therapeutic value of dual targeting of the immune and angiogenic pathways in managing advanced RCC. Continued investigation into biomarkers, patient selection, and optimized supportive care will further enhance the benefits of this combination therapy in the broader mRCC population.

## Author Contributions

Both authors contributed to the manuscript and were involved in providing case data and reviewing the manuscript.

## Funding

The authors received no specific funding for this work.

## Consent

Written informed consent was obtained from the patient(s) for publication of this case report and any accompanying images. A copy of the written consents is available for review by the editor in chief of this journal on request.

## Conflicts of Interest

The authors declare no conflicts of interest.

## Data Availability

The original contributions presented in the study are included in the article; further inquiries can be directed to the corresponding author.
